# Recurrent Abdominal Pain in a Premenarchal Girl: A Missed Diagnosis of Hematocolpos Identified by Point-of-Care Ultrasound

**DOI:** 10.7759/cureus.108169

**Published:** 2026-05-03

**Authors:** Omar Al Awni, Sohail S Alshahrani

**Affiliations:** 1 Pediatric Emergency Medicine, Security Forces Hospital, Riyadh, SAU

**Keywords:** emergency department, hematocolpos, imperforate hymen, pediatric abdominal pain, point-of-care ultrasound, primary amenorrhea

## Abstract

Hematocolpos is a rare but clinically significant cause of abdominal pain in peripubertal females and may be misdiagnosed as more common conditions such as constipation. We report a case of a girl aged 10 years and 10 months with multiple emergency department visits for recurrent abdominal pain, initially attributed to constipation with unremarkable prior imaging. She presented again with persistent symptoms and no history of menarche despite signs of puberty. Physical examination revealed findings suggestive of hematocolpos. Point-of-care ultrasound (POCUS) demonstrated a distended uterus, which was confirmed on formal ultrasonography. The patient was diagnosed with hematocolpos, likely secondary to an imperforate hymen, and underwent hymenectomy with a good outcome. This case highlights the importance of considering gynecologic etiologies in peripubertal females with abdominal pain and the utility of POCUS in early diagnosis.

## Introduction

Hematocolpos is an uncommon but clinically significant cause of abdominal pain in peripubertal females, most commonly resulting from outflow obstruction due to congenital anomalies such as an imperforate hymen. An imperforate hymen is a congenital condition characterized by failure of hymenal perforation, leading to obstruction of menstrual flow. Although well described, hematocolpos is frequently underdiagnosed due to its nonspecific clinical presentation, often mimicking more common conditions such as gastrointestinal or urinary disorders, which may delay diagnosis and increase the risk of complications [1,2].

In the pediatric emergency setting, early recognition of gynecologic causes of abdominal pain is essential. As outlined in Fleisher & Ludwig's Textbook of Pediatric Emergency Medicine, 8th Edition, abdominal pain in children requires a broad differential diagnosis with attention to age- and sex-specific etiologies [3]. In peripubertal females, obstructive reproductive tract anomalies should be considered, particularly in patients presenting with recurrent abdominal pain, abdominal distension, or urinary symptoms.

Point-of-care ultrasound (POCUS) has emerged as a valuable bedside diagnostic tool in pediatric emergency medicine, allowing rapid identification of pelvic pathology and facilitating timely management. We report a case of hematocolpos in a premenarchal girl presenting with recurrent abdominal pain, initially misdiagnosed as constipation, in which POCUS enabled early diagnosis and appropriate management.

## Case presentation

A previously healthy premenarchal peripubertal female presented to the emergency department with recurrent lower abdominal pain of several weeks’ duration. The pain was intermittent, progressively worsening in severity, and associated with abdominal distension. She had been evaluated previously and treated for presumed constipation without significant improvement.

There was no history of fever, vomiting, urinary symptoms, or prior abdominal surgery, and menarche had not yet occurred.

On examination, the patient appeared uncomfortable but was hemodynamically stable. Abdominal examination revealed a distended lower abdomen with suprapubic tenderness. No peritoneal signs were present. External genital examination was initially deferred due to patient discomfort and the clinical setting.

**Figure 1:**
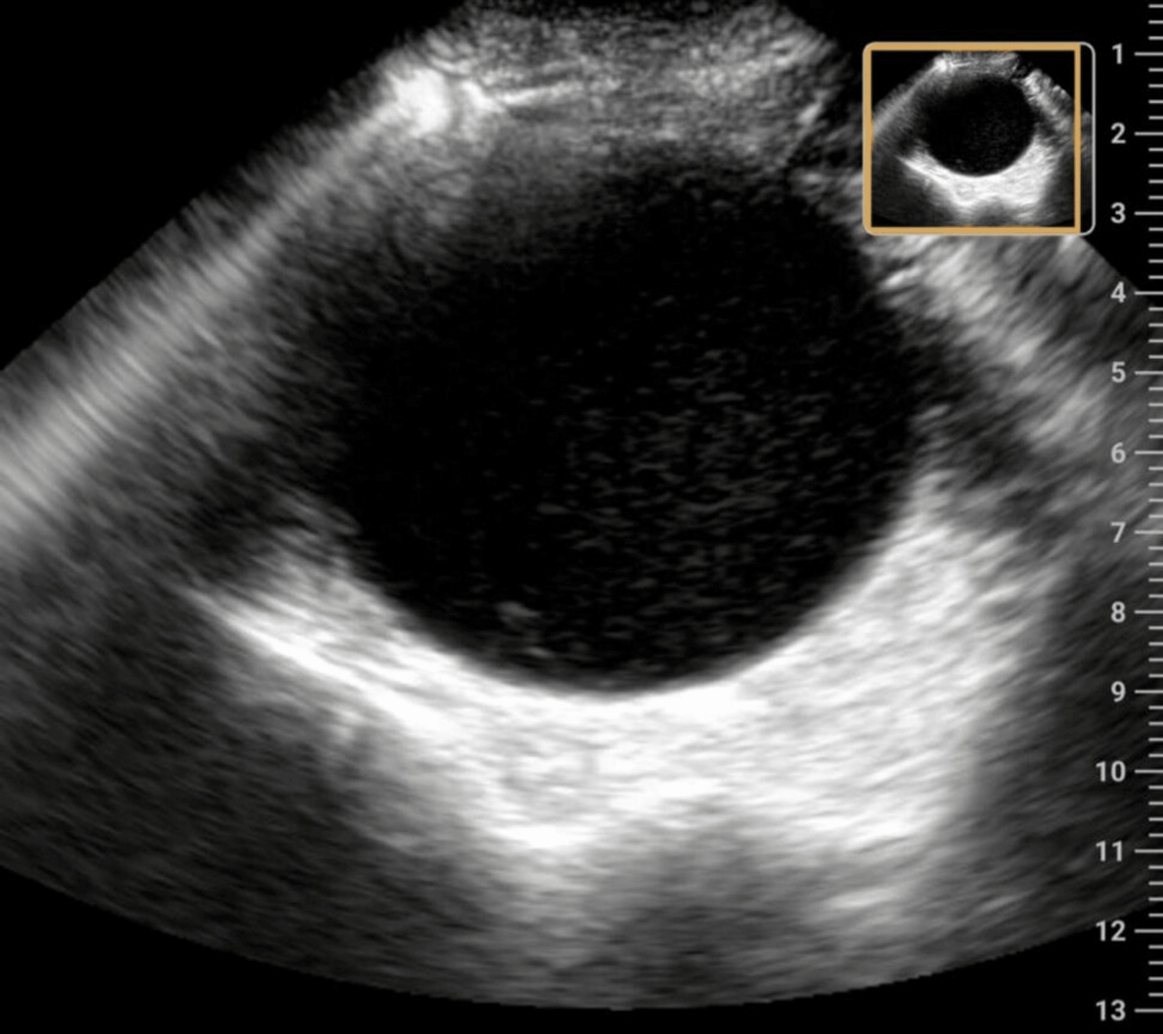
Point-of-care ultrasound demonstrating hematocolpos.

POCUS performed in the emergency department demonstrated a large, well-defined hypoechoic fluid collection within the pelvis, consistent with a distended vagina and uterus containing internal echoes suggestive of retained blood products (Figure 1). The urinary bladder was noted to be compressed anteriorly.

**Figure 2:**
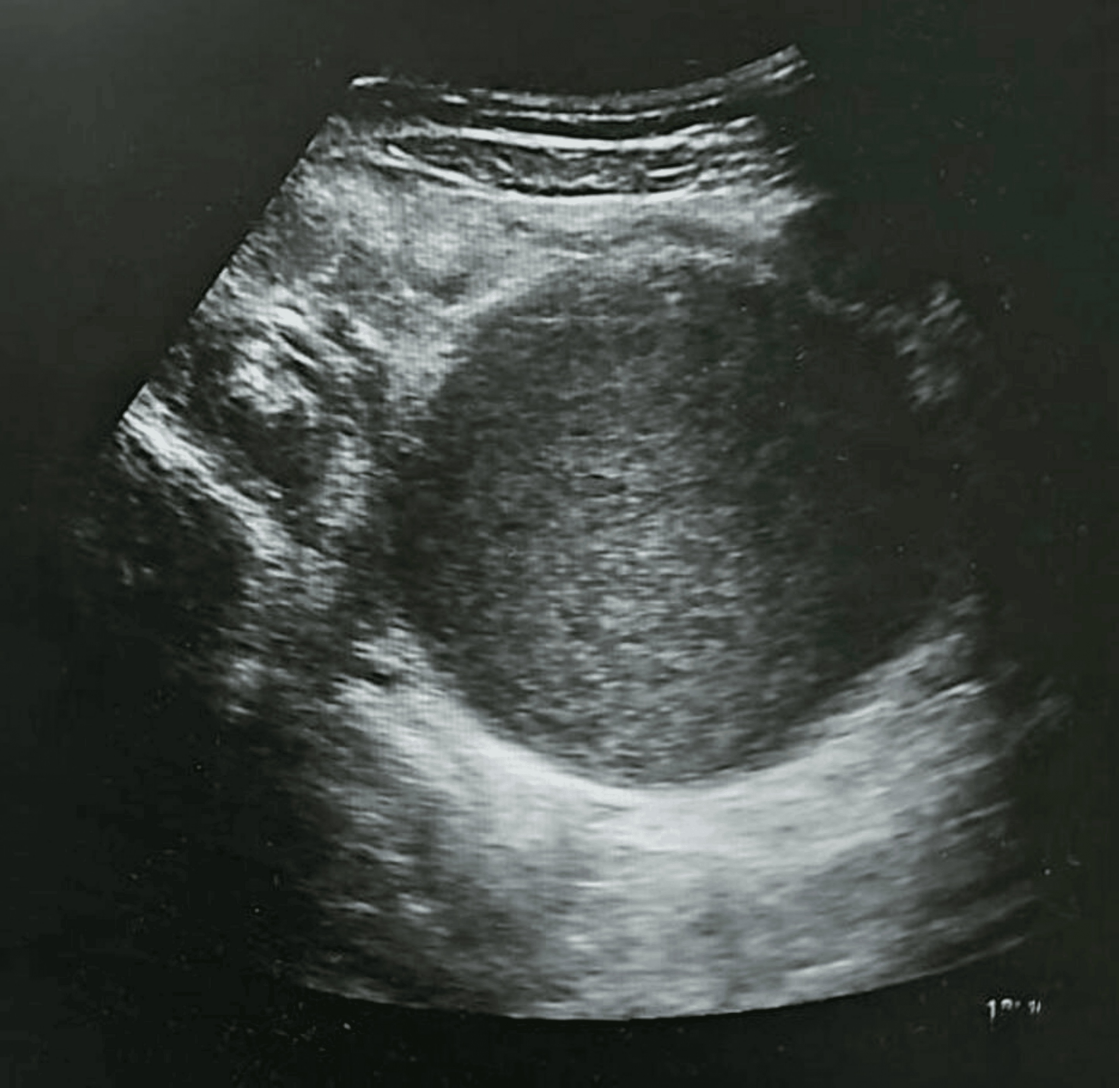
Pelvic point-of-care ultrasound demonstrating a distended, fluid-filled vagina and uterus consistent with hematocolpos.

Further evaluation confirmed the diagnosis of hematocolpos secondary to an imperforate hymen. These findings were corroborated on formal imaging (Figure 2). Gynecology was consulted, and given the patient’s age (<12 years), she was referred for definitive surgical management in accordance with institutional practice. The patient subsequently underwent hymenotomy with evacuation of retained menstrual blood.

The patient had an uneventful postoperative recovery, with complete resolution of symptoms on follow-up.

## Discussion

Hematocolpos is a recognized but frequently underdiagnosed cause of abdominal pain in peripubertal females, most commonly resulting from outflow obstruction due to an imperforate hymen. Its nonspecific presentation often leads to misdiagnosis as gastrointestinal or urinary conditions, contributing to delays in diagnosis and an increased risk of complications [1,2].

In the pediatric emergency setting, early recognition of gynecologic etiologies is essential. As emphasized in Fleisher & Ludwig's Textbook of Pediatric Emergency Medicine, 8th Edition, abdominal pain in children requires a broad differential diagnosis with careful consideration of age- and sex-specific causes [3]. In peripubertal females, obstructive reproductive tract anomalies should be considered, particularly in patients presenting with recurrent abdominal pain, abdominal distension, or urinary symptoms.

An imperforate hymen is the most common obstructive anomaly of the female genital tract and typically presents after menarche with primary amenorrhea and cyclic abdominal pain. However, earlier presentations prior to menarche, as demonstrated in this case, have been reported and may increase the likelihood of misdiagnosis [2,3]. Accumulation of menstrual blood within the vagina (hematocolpos), and occasionally extending to the uterus (hematometrocolpos), can result in a mass effect causing urinary retention, constipation, or pelvic discomfort.

POCUS has emerged as a valuable diagnostic tool in the emergency department. Previous studies have demonstrated that hematocolpos appears as a distended, fluid-filled vagina and uterus with internal echoes representing retained blood products [3,4]. In our case, bedside ultrasound facilitated early diagnosis, consistent with prior reports highlighting its role in reducing diagnostic delays and avoiding unnecessary investigations. Compared with formal ultrasound or MRI, POCUS provides a rapid, non-invasive, and readily available bedside modality, particularly useful in time-sensitive emergency settings.

Delayed diagnosis of hematocolpos has been associated with complications such as infection, endometriosis, hydronephrosis, and, in severe cases, infertility [2,4]. Our findings are consistent with the existing literature, reinforcing that early recognition and timely intervention can prevent these adverse outcomes. This case also underscores the importance of reconsidering the diagnosis in patients with persistent or recurrent symptoms despite standard treatment, such as presumed constipation.

Definitive management involves surgical correction, most commonly hymenotomy, which allows drainage of retained blood and resolution of symptoms. As reported in previous studies, prognosis is excellent when timely intervention is performed, consistent with the favorable outcome observed in our patient [1,3].

## Conclusions

Hematocolpos should be considered in premenarchal and peripubertal females presenting with recurrent abdominal pain and abdominal distension, particularly when symptoms are unexplained or refractory to initial treatment. Early use of POCUS in the emergency department can facilitate prompt diagnosis and help prevent complications associated with delayed recognition.

This case highlights the importance of maintaining a broad differential diagnosis and considering gynecologic etiologies in young patients with atypical or persistent abdominal symptoms.
